# Reduced vitamin D-induced cathelicidin production and killing of *Mycobacterium tuberculosis* in macrophages from a patient with a non-functional vitamin D receptor: A case report

**DOI:** 10.3389/fimmu.2022.1038960

**Published:** 2022-11-03

**Authors:** Fatima A. H. Al-Jaberi, Cornelia Geisler Crone, Thomas Lindenstrøm, Nicolai Skovbjerg Arildsen, Emilia Sæderup Lindeløv, Louise Aagaard, Eva Gravesen, Rasmus Mortensen, Aase Bengaard Andersen, Klaus Olgaard, Jessica Xin Hjaltelin, Søren Brunak, Charlotte Menné Bonefeld, Martin Kongsbak-Wismann, Carsten Geisler

**Affiliations:** ^1^ The LEO Foundation Skin Immunology Research Center, Department of Immunology and Microbiology, Faculty of Health and Medical Sciences, University of Copenhagen, Copenhagen, Denmark; ^2^ Centre of Excellence for Health, Immunity and Infections (CHIP), Rigshospitalet and Faculty of Health and Medical Sciences, University of Copenhagen, Copenhagen, Denmark; ^3^ Department of infectious disease immunology, Statens Serum Institut, Copenhagen, Denmark; ^4^ Department of Nephrology, University of Copenhagen, Rigshospitalet and Faculty of Health and Medical Sciences, Copenhagen, Denmark; ^5^ Novo Nordisk Foundation Center for Protein Research, University of Copenhagen, Faculty of Health and Medical Sciences, Copenhagen, Denmark

**Keywords:** tuberculosis, hereditary vitamin D-resistant rickets (HVDRR), macrophage, cathelicidin, vitamin D

## Abstract

Tuberculosis (TB) presents a serious health problem with approximately a quarter of the world’s population infected with *Mycobacterium tuberculosis* (*M. tuberculosis*) in an asymptomatic latent state of which 5–10% develops active TB at some point in their lives. The antimicrobial protein cathelicidin has broad antimicrobial activity towards viruses and bacteria including *M. tuberculosis*. Vitamin D increases the expression of cathelicidin in many cell types including macrophages, and it has been suggested that the vitamin D-mediated antimicrobial activity against *M. tuberculosis* is dependent on the induction of cathelicidin. However, unraveling the immunoregulatory effects of vitamin D in humans is hampered by the lack of suitable experimental models. We have previously described a family in which members suffer from hereditary vitamin D-resistant rickets (HVDRR). The family carry a mutation in the DNA-binding domain of the vitamin D receptor (VDR). This mutation leads to a non-functional VDR, meaning that vitamin D cannot exert its effect in family members homozygous for the mutation. Studies of HVDRR patients open unique possibilities to gain insight in the immunoregulatory roles of vitamin D in humans. Here we describe the impaired ability of macrophages to produce cathelicidin in a HVDRR patient, who in her adolescence suffered from extrapulmonary TB. The present case is a rare experiment of nature, which illustrates the importance of vitamin D in the pathophysiology of combating *M. tuberculosis*.

## Introduction

Tuberculosis (TB) presents a serious health problem with approximately 10 million new cases of active TB responsible for 1.5 million deaths in 2020 (1). The disease is caused by the bacteria *Mycobacterium tuberculosis* (*M. tuberculosis*). It is presumed that approximately a quarter of the world’s population is infected with *M. tuberculosis* in an asymptomatic latent state and that 5–10% of infected individual develop active TB at some point in their lives (2). *M. tuberculosis* is transmitted through aerosol droplets to the lung alveoli, where it infects alveolar macrophages and subsequently dendritic cells, neutrophils and macrophages in the lung interstitium (3). In the latent state, *M. tuberculosis* is contained by the immune system in granulomas consisting mainly of infected macrophages surrounded by T helper 1 (Th1) cells producing interferon-γ (IFNγ) (2, 4). Cathelicidin plays an important role in the ability of macrophages to kill bacteria, and it has been reported that IFNγ increases the expression of cathelicidin in monocytes and macrophages (5–7). Importantly, it was found that vitamin D is required for IFNγ-mediated enhancement of cathelicidin. This is in agreement with studies showing that vitamin D deficiency is associated with impaired expression of cathelicidin and increased susceptibility to some infectious diseases, including TB (8–15). This could also be an important mechanism explaining the beneficial role of vitamin D in TB prevention and treatment (16, 17).

Cathelicidins are a family of antimicrobial proteins. In humans, the cationic antimicrobial protein hCAP18 is the only cathelicidin identified to date (18). The antibacterial C-terminus of cathelicidin, LL37, has broad antimicrobial activity towards microorganisms including *M. tuberculosis* (19, 20). Cathelicidin is encoded by the gene *CAMP*, which is transcriptionally regulated by several transcription factors, including the VDR (21, 22). Cathelicidin is expressed by several immune cells and tissues that are exposed to microbes including the skin, the digestive tract and airways, and it circulates at high levels in the plasma (23, 24). Some studies have indicated that plasma cathelicidin levels correlate with plasma vitamin D levels in subjects with 25(OH)D concentrations ≤ 80 nM (12, 13). Others and we have demonstrated that vitamin D counteracts *M. tuberculosis*-induced cathelicidin down-regulation in dendritic cells and monocytes (25, 26), and it has been suggested that the vitamin D-mediated antimicrobial activity against *M. tuberculosis* is dependent on the induction of cathelicidin (27). Accordingly, many studies have indicated that vitamin D has a significant impact on immune responses *in vitro* (28–31). However, it is not known how vitamin D affects human immune responses *in vivo*, and studies of the immunoregulatory effects of vitamin D in humans is hampered by the lack of suitable experimental models. Hence, the role of vitamin D in mice cannot be directly extrapolated to humans as several vitamin D target genes are differently regulated in mice and humans (32, 33). Instead, we propose that the immunoregulatory roles of vitamin D could be determined by studying human subjects with a non-functional vitamin D receptor (VDR). Hereditary vitamin D-resistant rickets (HVDRR, OMIM 277440) is a rare autosomal recessive disorder, where the VDR is defective due to mutations in the gene encoding the VDR (34). Approximately 150 cases of HVDRR have been described (35), but only few preliminary studies on how the VDR mutations in these patients affect immune responses have been published (36–38).

Here we describe a HVDRR patient who suffered from extrapulmonary TB in her adolescence, and we demonstrate the impaired ability of her macrophages to produce cathelicidin and to combat *ex vivo* infection with *M. tuberculosis* in response to vitamin D.

## Case description

The patient was born in 1992 in Iraq to consanguineous parents. Within the first year after her birth, she developed rickets and alopecia. Test results from her time in Iraq are not available. In 1998, the family moved to Denmark, and in 1999 at the age of 7 years, the patient was referred to hospital due to muscle and bone pains, short stature and alopecia. The patient appeared normal except for alopecia and short stature with a height and weight below the 3% percentile. She had normal serum levels of calcium, phosphate, alkaline phosphatase and parathyroid hormone. However, at several measurements, the level of serum 1,25(OH)_2_D was highly elevated between 320 - 388 pM (normal range 51 – 177 pM). The patient was suspected of HVDRR based on the clinical findings of alopecia, delayed bone age and elevated levels of serum 1,25(OH)_2_D. Treatment with calcium and calcitriol was initiated. Further details of the case history have previously been published (39). The patient was homozygous for a missense mutation located in exon 3 of the VDR gene that causes exchange of arginine (R) to tryptophan (W) at position 80 (VDR^R80W^) in the second zinc-finger of the DNA binding domain of the VDR. The transcriptional activity of the VDR was abolished by the mutation. For further details of the molecular and functional characterization of the VDR mutation please see (39). In the years following the HVDRR diagnosis, the patient continued to have alopecia but met adequate growth. Calcitriol supplements were taken with varying compliance.

At the age of 16 years, the patient presented with vomiting and weight loss. She described 2 months of abdominal pain, vomiting 1-2 times daily and an unintended 5 kg weight loss. Upon admission, physical examination showed diffuse abdominal pain, temperature 39° Celsius and elevated C-reactive protein (71 mg/l). Empiric antibiotic treatment was initiated (tablet ampicillin 500 mg four time daily). Ultrasound examination of the abdomen showed ascites and possible carcinosis in form of solid tissue deposited on the right ovary and on the intestinal loops. Microscopy of the ascites fluid showed bleeding and lymphohistiocytosis, but culture and polymerase chain reaction test for tuberculosis were negative. Computed tomography (CT) of the chest and abdomen showed a cyst in the thyroid gland, ascites, carcinosis in the small pelvis and enlarged ovary, spleen and lymph nodes in relation to the right iliac artery. Gastroscopic examination and biopsies from duodenum and ventricle were performed without abnormal findings. Needle biopsies from peritoneum and ovary showed granulomatous inflammation with giant cells. Biopsies were without sign of malignancy and stains for tuberculosis, fungi and atypical mycobacteria were negative. Mantoux test showed no induration but redness of 0.7 cm in diameter. An *M. tuberculosis*-specific IFNγ release assay (IGRA) was performed with positive result and gastrointestinal tuberculosis was suspected. Three feces samples were examined for tuberculosis by culture without positive findings and the patient had a normal chest x-ray. Based upon the suspicion of TB, standard anti-tuberculosis treatment was initiated with rifampicin, isoniazid, pyrazinamide and ethambutol plus pyridoxin. The patient responded well to the treatment with normalization of C-reactive protein, reduction in the gastrointestinal symptoms and a slow weight gain. Controls with CT of the abdomen showed regression of the enlarged spleen, ovary and the necrotic lymph nodes, and the patient was reduced to maintenance treatment with isoniazid/pyridoxin and rifampicin after two months. After a total of 6 months treatment, the patient had normalized her weight and the gastrointestinal symptoms had resolved. No recognized exposure for TB was found, as none of the patient’s family had a history of tuberculosis and all members had normal findings on chest x-ray when examined in relation to the patient’s disease. The patient had not received Bacillus Calmette–Guérin (BCG) vaccine at any time.

At the time of inclusion in the present study, the patient was 27 years old, her heterozygous siblings between 31 and 37 years old, her parents 63 and 65 years old, and the control group (5 women and 8 men) between 20 and 62 years old. As previously described for many HVDRR patients with a non-functional VDR (36), the patient in the present study was able to maintain normal serum levels of calcium, parathyroid hormone and 1,25(OH)_2_D after puberty and into adulthood with modest or even without calcium supplements. The study was approved by The Committees of Biomedical Research Ethics for the Capital Region in Denmark (H-170409222). Written consent was obtained from all test subjects in accordance with the Declarations of Helsinki principles for research involving human subjects. For description of materials and methods, please see Supplementary Materials.

## Results and discussion

### Plasma cathelicidin levels are independent of VDR function and do not correlate to the levels of 25(OH)D or 1,_2_5(OH)2D 

Denmark is a low burden TB country with a TB incidence of approximately 5 per 100,000 population. However, the HVDRR patient was born in Iraq in 1992 and moved from Iraq to Denmark in 1998, at which time Iraq was a high burden country with a TB incidence rate of more than 40 per 100,000 population. It has been shown that immigrants from high-incidence countries can reactivate a latent TB many years after arrival to low-incidence countries (40). As no recognized exposure for TB in Denmark was found, we presume that the extrapulmonary TB of the HVDRR patient was caused by reactivation of a latent TB infection obtained in Iraq. However, we cannot prove this as no *M. tuberculosis* bacteria was isolated. To confirm that the HVDRR patient had mounted a specific immune response against *M. tuberculosis*, we measured the presence of *M. tuberculosis*-specific memory T cells with the IGRA QuantiFERON-TB Gold Plus. As expected, we found a strong reaction in the HVDRR patient. In contrast, *M. tuberculosis*-specific memory T cells were not detected in the controls and family members (**Supplementary Figure S1A**).

**Figure 1:**
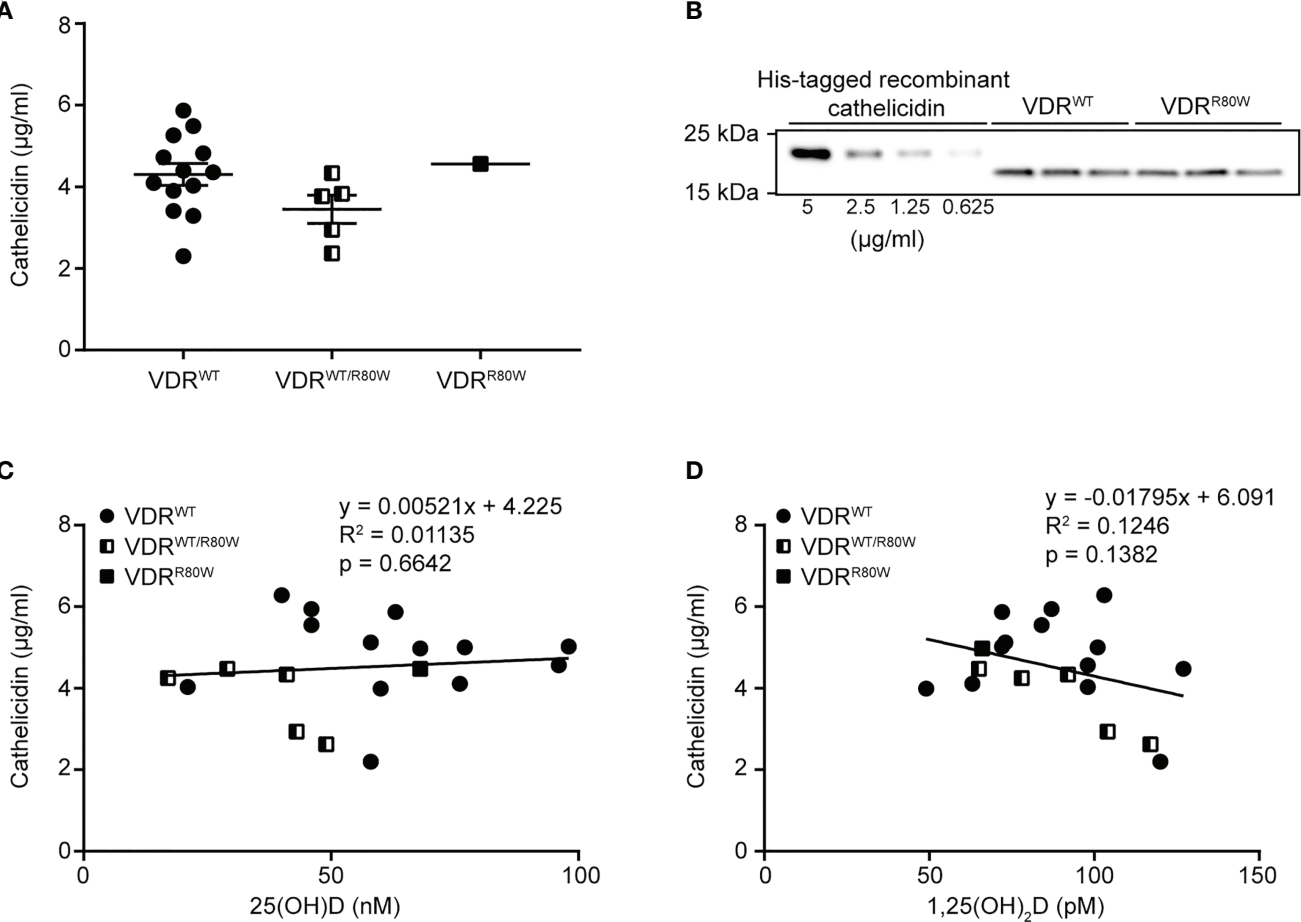
Plasma cathelicidin levels are independent of 25(OH)D and VDR function **(A)** Plasma cathelicidin levels in control subjects (VDR^WT^), heterozygous family members (VDR^WT/R80W^) and the HVDRR patient (VDR^R80W^). **(B)** Representative Western blot analysis of plasma cathelicidin from one control subject (VDR^WT^) and the HVDRR patient (VDR^R80W^) both run in triplicates. A titration of His-tagged recombinant cathelicidin used for the standard curve was included in each analysis. **(C)** Plasma cathelicidin versus 25(OH)D concentrations **(C)** or versus 1,25(OH)2D concentrations **(D)** in the control subjects (VDR^WT^), heterozygous family members (VDR^WT/R80W^) and the HVDRR patient (VDR^R80W^).

Whether a correlation between plasma levels of 25(OH)D and cathelicidin exists has been discussed for many years. Some studies have found a weak correlation (12, 13, 41), whereas other studies did not find a correlation (25, 42). In contrast, a strong correlation between the number of circulating neutrophils and the plasma level of cathelicidin has been described (43). If a correlation between the plasma levels of 25(OH)D and cathelicidin exists, this would mean that the plasma cathelicidin levels would be dependent on 1,25(OH)_2_D-mediated VDR signaling. To test whether VDR signaling affected the plasma levels of cathelicidin, we measured the levels of 25(OH)D, 1,25(OH)_2_D and cathelicidin in the plasma from healthy donors, family members heterozygous for the VDR^R80W^ mutation and from the HVDRR patient homozygous for the VDR^R80W^ mutation. We found that plasma cathelicidin levels were equal to our normal group in both the heterozygous carriers of the VDR^R80W^ mutation and the HVDRR patient (**Figures 1A, B**). As 1,25(OH)_2_D-mediated VDR signaling is severely compromised in the HVDRR patient and reduced by approximately 50% in the heterozygous carriers of the VDR^R80W^ mutation (39), these data demonstrated that 1,25(OH)_2_D-mediated VDR signaling does not affect the plasma levels of cathelicidin, at least in normal conditions. In addition, we did not find any correlation between the plasma levels of 25(OH)D and cathelicidin (**Figure 1C**) or 1,25(OH)_2_D and cathelicidin (**Figure 1D**), supporting that plasma cathelicidin levels are independent of 25(OH)D, 1,25(OH)2D and VDR function.

### Normal VDR expression levels and activation of macrophages from the HVDRR patient

**Figure 2:**
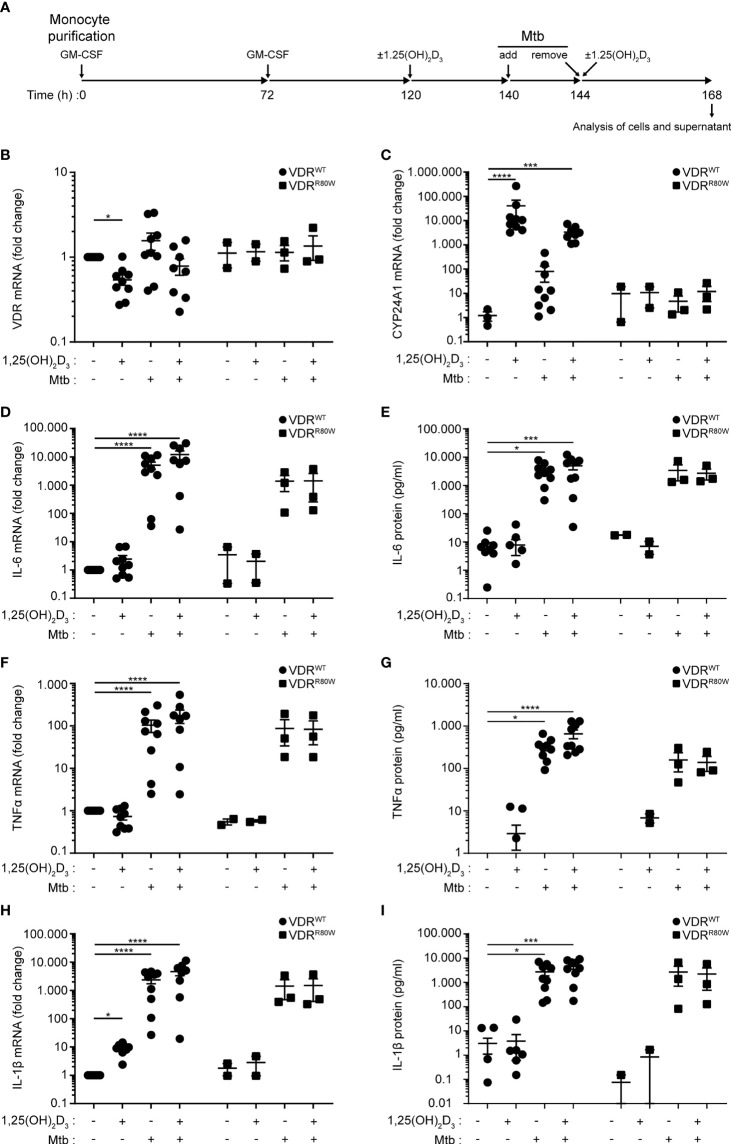
Normal VDR expression levels and activation of macrophages from the HVDRR patient **(A)** Overview of the experimental setup; Mtb (*M. tuberculosis*). **(B)** VDR, **(C)** CYP24A1, **(D)** IL-6, **(F)** TNFα and **(H)** IL-1β mRNA in macrophages and **(E)** IL-6, **(G)** TNFα and **(I)** IL-1β protein in the supernatants harvested at 168 h from control subjects (VDR^WT^) and the HVDRR patient (VDR^R80W^). The macrophages were treated with 1,25(OH)_2_D_3_ and *M. tuberculosis* as indicated below the graphs. **(B, D, F, H)** The expression levels of the indicated targets were normalized to the levels in untreated macrophages from control subjects. **(C)** CYP24A1 levels were normalized to the mean CYP24A1 level in untreated macrophages from control subjects. **(B–I)** Data from three independent experiments each with macrophages from three control subjects and the HVDRR patient. *p < 0.05; ***p < 0.001; ****p < 0.0001.

Previously, we have shown that the mutated VDR^R80W^ is expressed in T cells from the HVDRR patient at similar levels as the wild-type VDR in T cells from control subjects (39). To determine the effect of vitamin D and *M. tuberculosis* infection on the expression levels and function of the VDR in macrophages, we isolated monocytes from blood samples from controls and the HVDRR patient and differentiated them to macrophages with GM-CSF as outlined in **Figure 2A**. At 120 h, the macrophages were either left untreated or were treated with the active form of vitamin D, 1,25(OH)_2_D_3_. At 140 h, the macrophages were either supplemented with pure medium or with medium containing *M. tuberculosis* at a multiplicity of infection (MOI) of 10 to allow for infection of the macrophages. Following 4 hours of incubation, the medium was removed (144 h), and the infected and uninfected cells were re-supplemented with medium with and without 1,25(OH)_2_D_3_. Following an overnight incubation, cells and supernatants were harvested and analyzed (168 h). The mutated VDR^R80W^ was expressed at similar levels in macrophages from the HVDRR patient as in the controls, and *M. tuberculosis* infection did not affect the expression levels of the VDR gene significantly (**Figure 2B**). 1,25(OH)_2_D_3_ strongly induced CYP24A1 expression, which is upregulated downstream of VDR signaling (44), in control macrophages but not in macrophages from the HVDRR patient, confirming the loss of function of the mutated VDR^R80W^ (**Figure 2C**).

To study whether the mutated VDR^R80W^ affected general activation of the macrophages, we measured the levels of IL-6, TNFα and IL-1β in the cells and supernatant by RT-qPCR and ELISA at 168 h. We found that *M. tuberculosis* clearly activated macrophages from both controls and the HVDRR patient, and that 1,25(OH)_2_D_3_ did not affect the tested activation parameters, except for IL-1β that was significantly up-regulated by 1,25(OH)_2_D_3_ in the controls as previously described (45) (**Figures 2D-I**). Likewise, the mutated VDR^R80W^ did not seem to affect activation-induced expression of CD40 and CD80 (**Supplementary Figures S1B, C**). Taken together, these data showed that the mutated VDR^R80W^ is expressed at comparable levels in macrophages from the HVDRR patient as the wild-type VDR in macrophages from the controls, but that the VDR^R80W^ is non-functional. However, macrophages from the HVDRR patient were activated to the same extend by *M. tuberculosis* as macrophages from control subjects, indicating that VDR signaling was not essential for macrophage activation by *M. tuberculosis*

### Reduced vitamin D-induced cathelicidin expression and *M. tuberculosis* elimination in macrophages from the HVDRR patient

**Figure 3:**
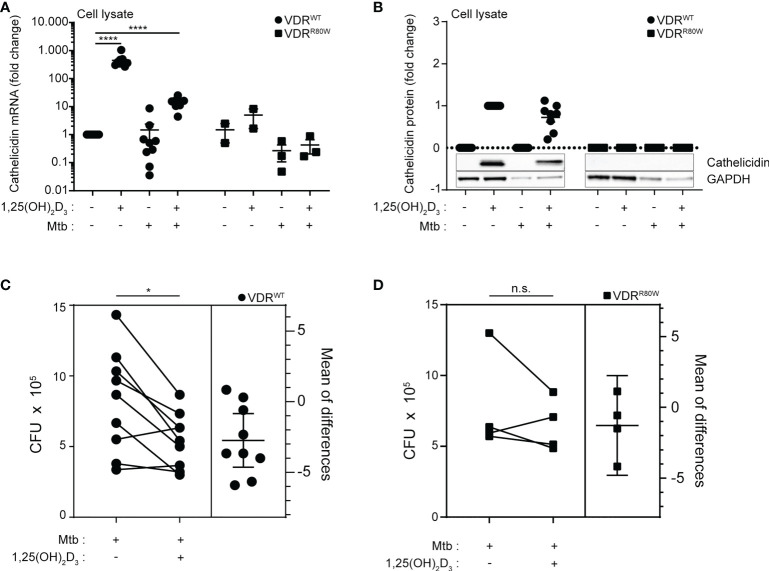
Reduced vitamin D-induced cathelicidin expression and *M. tuberculosis* elimination in macrophages from the HVDRR patient **(A)** mRNA and **(B)** protein levels of cathelicidin in macrophages from control subjects (VDR^WT^) and the HVDRR patient (VDR^R80W^). The macrophages were treated with 1,25(OH)_2_D_3_ and *M. tuberculosis* as indicated below the graphs. The cathelicidin levels were normalized to the cathelicidin levels in **(A)** untreated and **(B)** 1,25(OH)_2_D_3_-treated control cells. **(C, D)** Estimation plots of the CFU levels in macrophages infected with *M. tuberculosis* in the absence and presence of vitamin D from control subjects (VDR^WT^) and the HVDRR patient (VDR^R80W^). **(A–D)** Data from three independent experiments each with macrophages from three control subjects and the HVDRR patient. ns: not significant, *p < 0.05; ****p < 0.0001.

Previous studies have demonstrated that 1,25(OH)_2_D_3_ signaling increases the expression of cathelicidin in several cell types including macrophages (6, 26, 46, 47). Furthermore, studies have demonstrated that *M. tuberculosis* down-regulates the expression of cathelicidin in monocytes/macrophages (20, 25) and that this down-regulation can be partially counteracted by 1,25(OH)_2_D_3_ (26). To test how cathelicidin expression in macrophages was affected by 1,25(OH)_2_D_3_ and *M. tuberculosis* infection, we measured cathelicidin expression in cells harvested at 168 h (**Figure 2A**). We found that the levels of cathelicidin mRNA in untreated macrophages were comparable between the controls and the HVDRR patient (**Figure 3A**). However, whereas 1,25(OH)_2_D_3_ and infection with *M. tuberculosis* did not significantly affect cathelicidin expression in macrophages from the HVDRR patient, 1,25(OH)_2_D_3_ strongly up-regulated cathelicidin expression in both non-infected and infected macrophages from the control subjects (**Figure 3A**). These findings were also reflected in the intracellular expression of cathelicidin protein. Intracellular cathelicidin was clearly demonstrated in 1,25(OH)_2_D_3_-treated macrophages from the control subjects by Western blot analyses but could not be detected in macrophages from the HVDRR patient (**Figure 3B**).

To investigate whether the reduced 1,25(OH)_2_D_3_-induced expression of cathelicidin in macrophages from the HVDRR patient resulted in impaired *in vitro* killing of *M. tuberculosis*, we determined the CFU in untreated and 1,25(OH)_2_D_3_-treated macrophages infected with *M. tuberculosis* from the HVDRR patient and control subjects. The CFU levels in macrophages from the HVDRR patient and the control subjects were similar in the absence of 1,25(OH)_2_D_3_. However, whereas 1,25(OH)_2_D_3_ significantly reduced CFU numbers in macrophages from the control subjects, it did not seem to have an effect on *M. tuberculosis* survival in macrophages from the HVDRR patient (**Figures 3C, D**). Taken together, these data indicated that the macrophages from the HVDRR patient had a reduced ability to kill *M. tuberculosis* at least in part due to the defect in 1,25(OH)_2_D_3_-induced cathelicidin production. We could not exclude that other mechanisms added to the reduced ability to eliminate *M. tuberculosis* in the macrophages from the HVDRR. Thus, it has been reported that DEFB4 and IL-1β play a role in combating *M. tuberculosis* and that both are regulated by 1,25(OH)_2_D_3_ (45, 48). In general, 1,25(OH)_2_D_3_ affects a wide panel of immune parameters *in vitro*, including T cell activation, differentiation and cytokine production (28–31). We cannot exclude that dysregulation of some of these parameters might have contributed to the development of extrapulmonary TB in the HVDRR patient.

In conclusion, the present case report describes a case of TB infection in a 16-year-old girl who was diagnosed with HVDRR due to a non-functional VDR. Macrophages from the patient showed impaired 1,25(OH)_2_D_3_-induced cathelicidin expression and killing of *M. tuberculosis*. As a case report with only one patient, this report has some inherent limitations and the conclusion would be strengthened by the inclusion of more patients. However, the patient described here is the only patient with HVDRR in Denmark. Thus, this case is a rare experiment of nature that points to the importance of vitamin D in the pathophysiology of combating *M. tuberculosis*.

## Data availability statement

The original contributions presented in the study are included in the article/**Supplementary Material**, further inquiries can be directed to the corresponding author/s.

## Ethics statement

The studies involving human participants were reviewed and approved by The Committees of Biomedical Research Ethics for the Capital Region in Denmark (H-170409222). The patients/participants provided their written informed consent to participate in this study.

## Author contributions

CG and MK-W conceived the study. CG, MK-W, TL and RM designed the experiments. MK-W, FA-J, TL, EL, LA and EG performed the laboratory experiments. CC and AA supplied the clinical data. CB, KO, NA, JH and SB and assisted with the experimental design and/or data interpretation. CG, CC, and MK-W analyzed the data and wrote the manuscript with input from all authors. All authors contributed to the article and approved the submitted version.

## Funding

This work was financially supported by grants from the Danish Council for Independent Research (8020-00066B).

## Conflict of interest

The authors declare that the research was conducted in the absence of any commercial or financial relationships that could be construed as a potential conflict of interest.

## Publisher’s note

All claims expressed in this article are solely those of the authors and do not necessarily represent those of their affiliated organizations, or those of the publisher, the editors and the reviewers. Any product that may be evaluated in this article, or claim that may be made by its manufacturer, is not guaranteed or endorsed by the publisher.
