# Supplementary material for: Reduced vitamin D-induced cathelicidin production and killing of Mycobacterium tuberculosis in macrophages from a patient with a non-functional vitamin D receptor: A case report
Source: Front Immunol. 2022 Nov 3;13:1038960. doi: 10.3389/fimmu.2022.1038960 (PMC9672840; doi:10.3389/fimmu.2022.1038960)
Supplement: Supplementary file 1 [file DataSheet_1.pdf]

Supplementary Materials

Reduced vitamin D-induced cathelicidin production and killing of *Myco-bacterium tuberculosis* in macrophages from a patient with a non-functional vitamin D receptor: A case report

Materials and methods

Chemicals and antibodies

$1,25(OH)_2D_3$ (Cat: BML-DM200-0050) was from Enzo Life Sciences, Inc., Ann Arbor, MI. Stock solutions of 2.4 mM $1,25(OH)_2D_3$ were prepared in anhydrous (>99.5%) ethanol. GM-CSF was from PEPROTECH, Inc. (Cat: AF-HDC, USA). Primary antibodies used in Western blotting analyses included anti-LL-37 (D-5) (SC-166770, Santa Cruz, CA, USA) and anti-GAPDH from (Ab9485, Abcam, Cambridge, MA). Recombinant human cathelicidin (ab140725) containing a N-terminal His-tag was from Abcam.

Production and infection of monocyte-derived macrophages

PBMCs were isolated from peripheral blood samples by Lymphoprep (Axis Shield from Oslo, Norway) density gradient centrifugation by using SepMate™ tubes (Cat: 85450, Stemcell Technologies, Canada). The subsequent isolation of monocytes from the mononuclear cell fraction were by using EasySep Monocyte Isolation kit (Cat: 19359, Stemcell Technolo-

gies). In short, PBMCs were supplemented with antibodies against unwanted cells, and subsequently bound with beads. Then, the PBMCs were incubated in EasySep magnet (Cat: 18000, Stemcell Technologies) where unwanted cells bound to magnetic particles were be withheld. Following monocyte purification, 300.000 cells/ml were cultured 48-well plates (Cat: 150687, Nunc) in RPMI-1640 medium (Cat: R5886, Sigma Aldrich) supplemented with 1% penicillin/streptomycin (Pen/Strep), 1%L-Glutamine, 10% heat-inactivated, endotoxin-free fetal bovine serum (FBS) (Cat: 10082-147, Gibco) and GM-CSF (50 ng/ml) for 120 hours. Following the first 72 hours, cells were supplemented with additional 0.5 ml GM-CSF-containing medium. After 120 h, the supernatant was removed and the cells were supplemented with fresh GM-CSF-containing medium without Pen/Strep and left untreated or treated with $1,25(OH)_2D_3$ for 20 hours. At 140 hours, samples were either mock-infected or infected with *M. tuberculosis* Erdman strain (multiplicity of infection (MOI) 10) in medium with HEPES and 10 % FCS for 4 h. The supernatants were harvested, and the cells were re-supplemented with fresh culture medium of RPMI with HEPES and 10% FCS The harvested supernatant was treated with gentamycin (100 ug/ml) (O/N, 4 ^{o}C) to kill the remaining live *M. tuberculosis*. The cells are either left untreated or further treated with $1,25(OH)_2D_3$ for 24 h. The supernatants were harvested and incubated with 2.5% sodium azide (NaN_3) for 24 h and used for cytokine measurements by ELISA. The cells were then divided in three groups. The first group of cells were treated with lysis buffer (50 mM Tris base, pH 7.5, 150 mM NaCl and 1 mM $MgCl_2$) supplemented with 1% (vol/vol) Triton X-100, 1 x Protease/phosphatase inhibition cocktail (Cat: 5872S, Cell Signaling Technologies), 5 mM EDTA and gentamycin) and used for measurement of cathelicidin protein levels by Western blotting analysis. The second group of cells were treated with TRI reagent (Cat: T9424, Sigma Aldrich) and used for measurement of mRNA levels by RT-qPCR. The third group of cells were lysed

in lysis buffer for 30 minutes, cooled down to -80°C and subsequently used for CFU counting by spot inoculation.

RT-qPCR

mRNA levels for various targets were measured by RT-qPCR. Following cell isolation, cells were lysed in TRI reagent (Cat: T9424, Sigma Aldrich) and mixed with phase separation reagent 1- bromo-3-chloropropane (Cat: B9673, Sigma Aldrich). The RNA phase was isolated and mixed with isopropanol supplemented with glycogen for RNA precipitation (Cat: 10814-010, Invitrogen). The RNA pellet was then washed in RNase free 75% ethanol 3 times. cDNA was synthesized from quantified RNA using High Capacity RNA-to-cDNA™ Kit (Cat: 4387406, Applied Biosystems) according to manufacturer's instructions. For RT-qPCR, 12.5 ng cDNA was mixed with TaqMan® Universal Master Mix II with Uracil-N glycosylase and the target primers. We used the following primers from Applied Biosystems: VDR (Hs01045840_m1), Cathelicidin/CAMP (Hs00189038_m1), CYP24A1 (Hs00167999_m1), GAPDH (Hs02786624_g1), IL-1β (Hs01555410_m1), CD40 (Hs01002913_g1), CD80 (Hs01045161_m1), TNF (Hs00174128_m1), IL-6 (Hs00174131_m1). The plate-based detection instrument LightCycler ® 480 II from Roche was used for real-time PCR amplification. Expression levels of target genes were set relative to expression levels of GAPDH.

Western blotting analysis

For Western blotting analysis, cells were lysed in lysis buffer (50 mM Tris base, pH 7.5, 150 mM NaCl and 1 mM $MgCl_2$) supplemented with 1% (vol/vol) Triton X-100, 1 x Protease/phosphatase inhibition cocktail (Cat: 5872S, Cell Signaling Technologies). For western blotting analysis of plasma, blood samples was collected in plasma preparation tubes (PPT)

(Cat: 362788, BD Biosciences) containing EDTA. Blood samples were immediately centrifuged for 10 min at 1.100G, and plasma was removed. Plasma was diluted 20 times in RPMI-1640 medium prior to gel electrophoresis. Diluted plasma or cell lysates were supplemented with NuPAGE LDS sample buffer (4x) (Cat: NP0007, ThermoFisher Scientific) and NuPAGE sample reducing agent (10x) (Cat: NP0004, ThermoFisher Scientific) and treated for 5 minutes at 90 ^{o}C . Subsequently, proteins were separated by electrophoresis through NuPAGE™ 10% BisTris gels (Cat: NP0302BOX or NP0301BOX, Life Technologies). Proteins were transferred to nitrocellulose membranes (Cat: LC2001, Life Technologies) and visualized with primary antibodies and HRP-conjugated rabbit anti-mouse Ig (Cat: P0260, DAKO) or HRP-conjugated swine anti-rabbit Ig (Cat: P0399, DAKO) with ECL luminescence reagent (Cat: RPN2232, Sigma Aldrich) on a ChemiDoc^{TM} MP Imaging System (Bio Rad) and subsequently analyzed using the software ImageLab.

QuantiFERON-TB Gold Plus (QFT-Plus)

For detection of *M. tuberculosis*-specific memory T cells, the IGRA QuantiFERON-TB Gold Plus (Cat: 622536, (QFT-Plus), QIAGEN, USA) was used. In short, blood samples were collected in the four tubes (Nil, TB1, TB2 and Mitogen) and inverted 10 times. The tubes were incubated at 37 ^{o}C over-night and the plasma was subsequently collected and analyzed for IFNγ.

ELISA

Cytokine concentrations were determined by ELISA using Ready-Set-Go kits (IL-6, 88-7066-88; TNF, 88-7346-88; IL-1β, 88-7261-88) according to the manufacturer's instruction.

Quantification and Statistical Analysis

Quantification of bands from Western blotting analysis was performed using the programs ImageLab (BioRad) and Fiji. Statistical analysis and graphical representation of all data was performed using Prism 7 (GraphPad Soft-ware) and Adobe Illustrator CS6 (Adobe Systems Incorporated). In Figure 1C and 1D, linear regression analyses were performed indicating no correlation between plasma 25(OH)D or plasma $25(OH)_2D$ and plasma cathelicidin levels. For data from control subjects in Fig. 2B-I, 3A and S1B-C, ordinary one-way ANOVA with post hoc multiple comparisons testing (Dunnett's) were performed, where the means were compared to unstimulated macrophages.
