# Supplementary material for: Reduced vitamin D-induced cathelicidin production and killing of Mycobacterium tuberculosis in macrophages from a patient with a non-functional vitamin D receptor: A case report
Source: Front Immunol. 2022 Nov 3;13:1038960. doi: 10.3389/fimmu.2022.1038960 (PMC9672840; doi:10.3389/fimmu.2022.1038960)
Supplement: Supplementary file 2 [file DataSheet_2.pdf]

Supplementary Figure 1

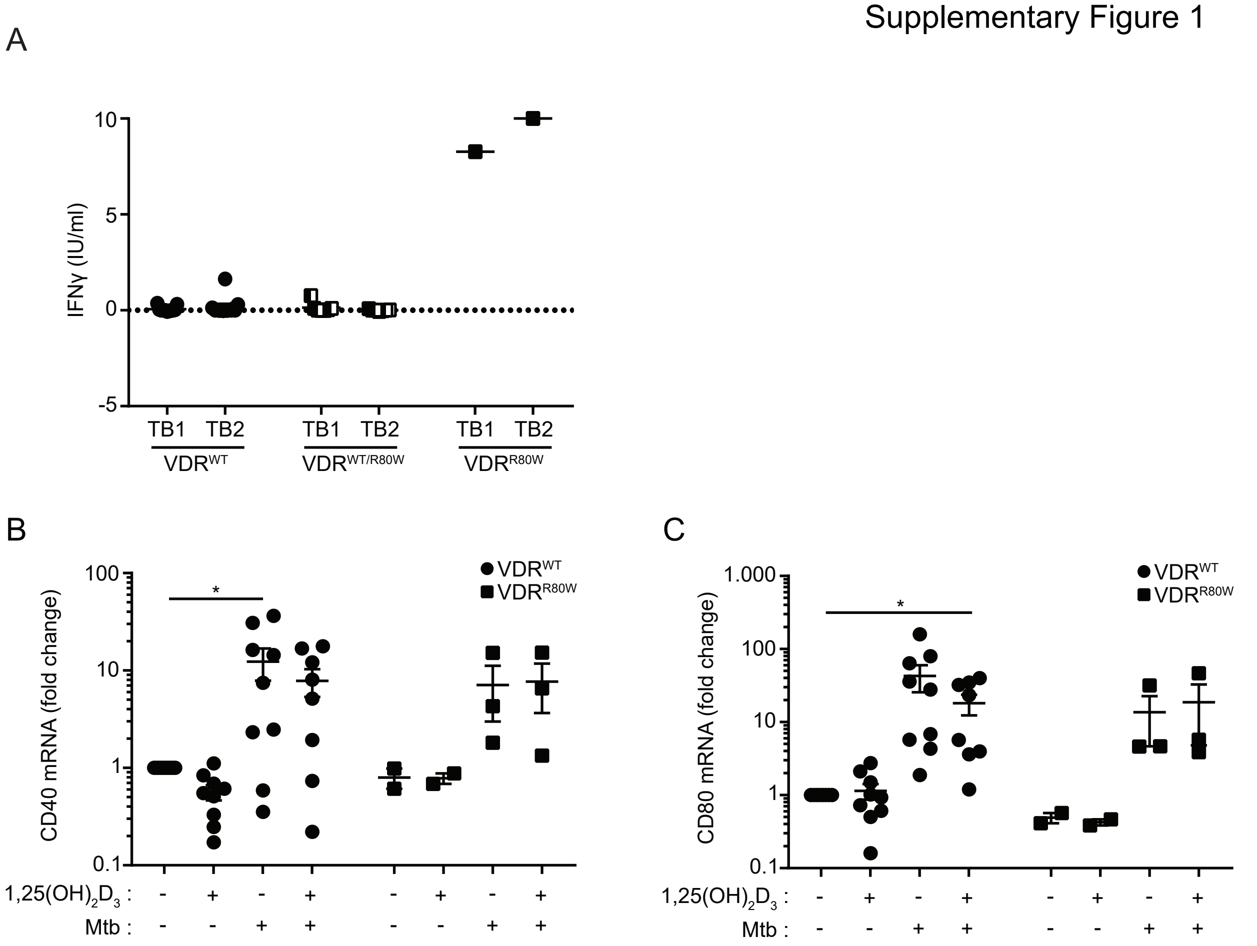

Supplementary Figure 1. IFNγ release and CD40 and CD80 expression

(A) IFNγ levels in the TB1 and TB2 tubes of the QuantiFERON-TB Gold Plus assay performed on blood samples from the control subjects (VDR^{WT}), heterozygous family members ($VDR^{WT/R80W}$) and the HVDRR patient (VDR^{R80W}). **(B)** CD40 and **(C)** CD80 mRNA in macrophages harvested at 168 h from control subjects (VDR^{WT}) and the HVDRR patient (VDR^{R80W}). The macrophages were treated with 1,25(OH)2D3 and *M. tuberculosis* as indicated below the graphs. The expression levels of the indicated targets were normalized to the levels in untreated macrophages from control subjects. **(B and C)** Data from three independent experiments each with macrophages from three control subjects and the HVDRR patient.
